# Dissecting Clinical and Metabolomics Associations of Left Atrial Phasic Function by Cardiac Magnetic Resonance Feature Tracking

**DOI:** 10.1038/s41598-018-26456-8

**Published:** 2018-05-25

**Authors:** Angela S. Koh, Fei Gao, Shuang Leng, Jean-Paul Kovalik, Xiaodan Zhao, Ru San Tan, Kevin Timothy Fridianto, Jianhong Ching, Serene JM Chua, Jian-Min Yuan, Woon-Puay Koh, Liang Zhong

**Affiliations:** 1 National Heart Centre Singapore, Singapore, Singapore; 2 Duke-NUS Medical School, Singapore, Singapore; 3 Department of Endocrinology, Singapore General Hospital, Singapore, Singapore; 4 Saw Swee Hock School of Public Health, National University of Singapore, Singapore, Singapore; 5 Division of Cancer Control and Population Sciences, University of Pittsburgh Cancer Institute, Pittsburgh, PA USA; 6 Department of Epidemiology, Graduate School of Public Health, University of Pittsburgh, Pittsburgh, PA USA

## Abstract

Among community cohorts, associations between clinical and metabolite factors and complex left atrial (LA) phasic function assessed by cardiac magnetic resonance (CMR) feature tracking (FT) are unknown. Longitudinal LA strain comprising reservoir strain (εs), conduit strain (εe) and booster strain (εa) and their corresponding peak strain rates (SRs, SRe, SRa) were measured using CMR FT. Targeted mass spectrometry measured 83 circulating metabolites in serum. Sparse Principal Component Analysis was used for data reduction. Among community adults (n = 128, 41% female) (mean age: 70.5 ± 11.6 years), age was significantly associated with εs (β = −0.30, p < 0.0001), εe (β = −0.3, p < 0.0001), SRs (β = −0.02, p < 0.0001), SRe (β = 0.04, p < 0.0001) and SRe/SRa (β = −0.01, p = 0.012). In contrast, heart rate was significantly associated with εa (β = 0.1, p = 0.001) and SRa (β = −0.02, p < 0.0001). Serine was significantly associated with εs (β = 10.1, p = 0.015), SRs (β = 0.5, p = 0.033) and SRa (β = −0.9, p = 0.016). Citrulline was associated with εs (β = −4.0, p = 0.016), εa (β = −3.4, p = 0.002) and SRa (β = 0.4, p = 0.019). Valine was associated with ratio of SRe:SRa (β = −0.4, p = 0.039). Medium and long chain dicarboxyl carnitines were associated with εs (β = −0.6, p = 0.038). Phases of LA function were differentially associated with clinical and metabolite factors. Metabolite signals may be used to advance mechanistic understanding of LA disease in future studies.

## Introduction

Historically, evaluation of left atrial (LA) function such as left atrial strain, strain rate and LA active or passive emptying fractions was performed using two-dimensional echocardiography techniques employing tissue Doppler imaging or speckle tracking methods^1,2^. More recently, studies have used cardiac magnetic resonance (CMR) feature tracking to characterize LA function since it allows more comprehensive assessment of complex LA phasic behaviour^3–5^. This technique has been used to assess left atrial phasic behaviour in clinical cohorts for risk stratification^6^ as well as for prognostication of incident cardiovascular events^7^. These developments suggest that left atrial phasic function investigations, in contrast to gross changes in left atrial size commonly used for risk stratification and prognostication, may be valuable for studying development of cardiovascular disease. Alterations in left atrial size are commonly associated with CVD risk factors such as hypertension and diabetes mellitus. However, there is little data on factors that influence left atrial phasic behaviour, particularly using community-based cohorts prior to development of overt cardiovascular disease. It is therefore unknown if underlying risk factors influence left atrial phasic behaviour prior to disease. Furthermore, while it has been reported that left atrial function may be significantly altered with aging^1^, no studies have included elderly adults primarily in their analysis^4^. The fact that cardiovascular disease (CVD) is a leading cause of death in older adults^8^, underscores the importance of gaining a better understanding of the impact of age (also an independent risk factor of CVD) on left atrial function as assessed by CMR feature tracking (FT).

Mounting data suggest that changes in fuel metabolism are associated with clinical CVD in both the general population^9–12^ as well as in elderly subjects^13^. In a study of patients with established cardiovascular disease, disturbances in the dicarboxyl/hydroxyl acyl-carnitine pathway were able predict incident cardiovascular events^12^. Furthermore, a distinct signature comprising of medium and long chain dicarboxyl and hydroxyl acyl-carnitines, likely reflecting changes in cellular fatty acid oxidation, was independently associated with arterial stiffness among aged adults without clinical cardiovascular disease^14^. Therefore, identification of distinct associations between metabolic perturbations with phases of left atrial function may therefore advance mechanistic understanding of how disordered metabolism drives left atrial diseases with aging, providing translatable knowledge for future therapeutics and/or preventative treatments.

In this study, we hypothesized that left atrial phasic function is related to clinical factors. We further hypothesize that changes in circulating metabolite profiles could be related to alterations in left atrial phasic function, independent of clinical factors. Therefore, we aimed to study the association between individual components of LA phasic function and clinical factors. Secondly, we aimed to characterize the relationship between metabolic profile of these subjects and components of left atrial function.

## Methods

The subjects were recruited from the Cardiac Aging Study (CAS), a prospective study initiated in 2014 that examines characteristics and determinants of cardiovascular function in elderly adults^14^.

The current analysis is a cross-sectional analysis between left atrial function and metabolomics profiling obtained from subjects recruited from the CAS study. Subjects who had self-reported history of physician-diagnosed cardiovascular disease (such as coronary heart disease, atrial fibrillation and stroke) or cancer were excluded. A total of 128 participants were studied in this analysis. The SingHealth Centralised Institutional Review Board had approved the study protocol. Informed consent was obtained from all participants. All methods were performed in accordance with the relevant guidelines and regulations.

All participants were examined and interviewed on one study visit by trained study coordinators. Participants completed a standardized questionnaire that included medical history and coronary risk factors. Hypertension was defined by current use of antihypertensive drugs or physician-diagnosed hypertension. Diabetes mellitus was defined by current use of anti-diabetic agents or physician-diagnosed diabetes mellitus. Dyslipidemia was defined by current use of lipid-lowering agents or physician-diagnosed dyslipidemia. Smoking history was defined as ever smokers (former or current smoking) or never smokers. Body mass index was calculated as weight in kilograms divided by the square of height in meters. Sinus rhythm status was ascertained by resting electrocardiogram. Clinical data were obtained on the same day as assessment of cardiac magnetic resonance (CMR) imaging and serum collection.

### CMR protocol and analysis

**Figure 1:**
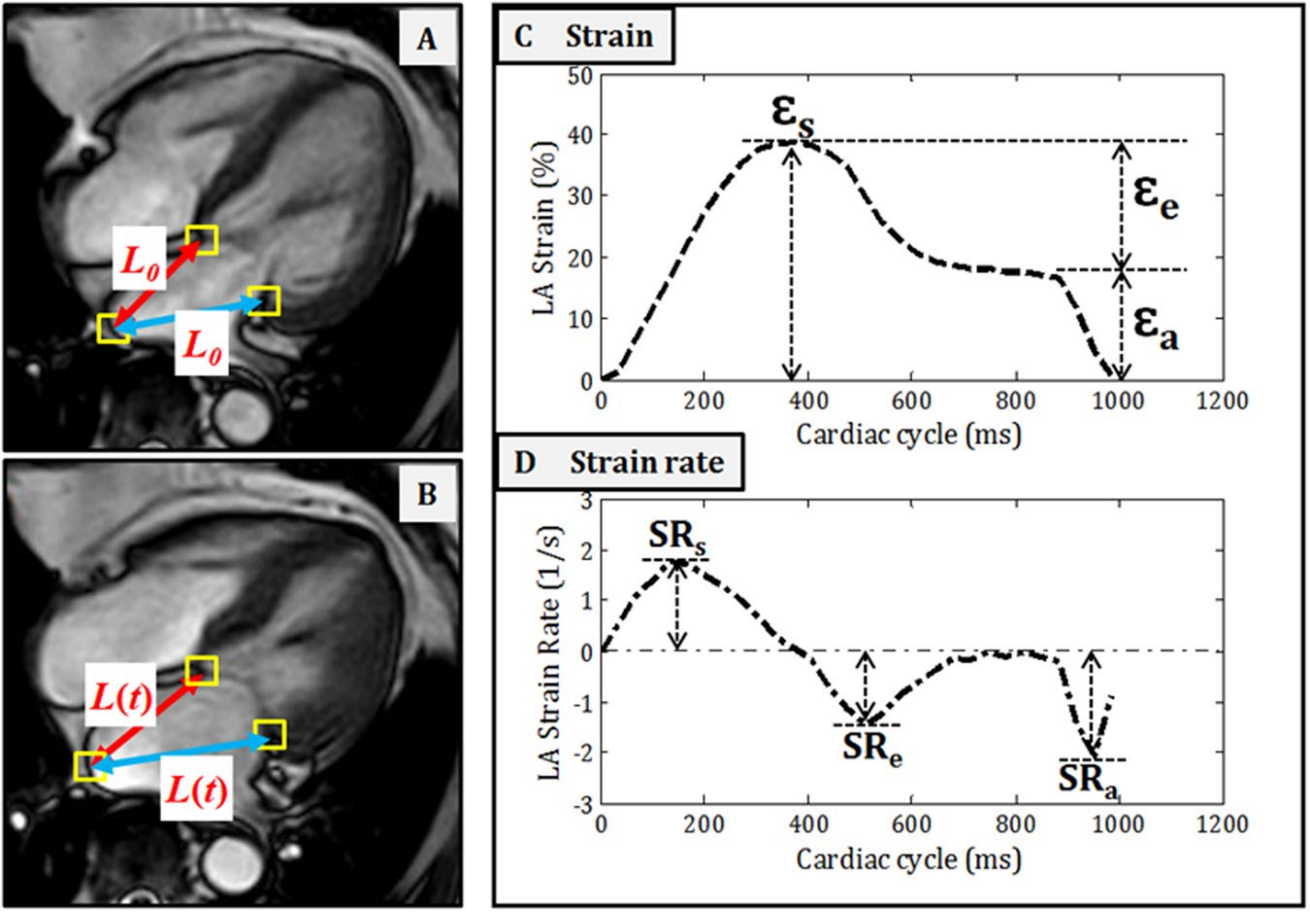
(**A**,**B**) semi-automatic algorithm to track the distance (L) between the left atrioventricular junction and a user-defined point at the mid posterior left atrial (LA) wall on standard CMR 2- and 4-chamber views. (**C**,**D**) Longitudinal strain (ε) at any time point (t) in the cardiac cycle from end-diastole (time 0) was calculated as: ε(t) = (L(t) − L_0_)/L_0_. LA reservoir strain (ε_s_), conduit strain (ε_e_) and booster strain (ε_a_) were calculated at *t* equals left ventricular end-systole, diastasis and pre-LA systole, respectively, and their corresponding peak strain rates (SR) derived.

Cine CMR scans were performed using balanced fast field echo sequence (BFFE). All subjects were imaged on a 3 T magnetic resonance imaging system (Ingenia, Philips Healthcare, The Netherlands) with a dStream Torso coil (maximal number of channels 32). BFFE end-expiratory breath hold cine images were acquired in multi-planar long-axis views (2-, 3-, and 4-chamber views). Typical parameters were as follows: TR/TE 3/1 ms; flip angle, 45°; in-plane spatial resolution, 1.0 mm × 1.0 mm to 1.5 mm × 1.5 mm; slice thickness, 8 mm; pixel bandwidth, 1797 Hz; field of view, 300 mm; frame rate, 30 or 40 per cardiac cycle. We developed an in-house semi-automatic algorithm to track the distance (L) between the left atrioventricular junction and a user-defined point at the mid posterior LA wall on standard CMR 2- and 4-chamber views. Both 2- and 4-chamber views were used to generate the average strain and strain rate results. Longitudinal strain (*ε*) at any time point (*t*) in the cardiac cycle from end-diastole (time 0) was calculated as: $\varepsilon (t)=(L(t)-{L}_{0})/{L}_{0}$. LA reservoir strain (*ε*_*s*_), conduit strain (*ε*_*e*_) and booster strain (*ε*_*a*_) were calculated at *t* equals left ventricular end-systole, diastasis and pre-LA systole, respectively, and their corresponding peak strain rates (SR) derived (Fig. 1). Strain and strain rate parameters from both 2- and 4-chamber views were averaged to obtain mean results for analysis. Using data from 20 randomly selected subjects, intra- and inter-observer comparability was assessed using Bland-Altman plot (Supplementary Fig. S1a,b). Two independent observers analyzed all cases in the evaluation of inter-observer variability (SL & XDZ), while intra-observer variability was assessed from a repeated analysis by the first observer (SL) after 7 days (Supplementary Fig. S1a,b). This technique has been validated against volumetric measurements and the strain results obtained from commercial software. Details about the technique can be found in the Supplementary Table S1 and Supplementary Fig. A.

### Central Hemodynamics

We measured central blood pressure parameters such as central systolic blood pressure, central diastolic blood pressure, central mean arterial pressure and central pulse pressure noninvasively using applanation tonometry (SphygmoCor system, AtCor Medical, Sydney, Australia). All measurements were performed in the daytime, in a quiet environment, at stable room temperature. Participants were studied in the supine position.

Antecubital venous blood samples (20–30 ml) were taken from consenting participants in the morning; fasting was not required before blood collection. After collection, the blood samples were immediately placed on ice for transportation and were processed within 6 h to obtain serum samples, which were subsequently stored at −80 °C.

Serum metabolomic profiling analysis was performed in the Duke-NUS Metabolomics Facility as previously described^15^. Thawed serum samples (100 μl) were spiked with 20 μl deuterium-labelled amino acid/acyl-carnitine mixture and diluted with 800 µl methanol. After centrifugation of the mixture at 17,000 g for 5 mins at 20 °C, the supernatant fraction was collected and divided into two parts: one (100 μl) for acylcarnitine analysis and one (10 μl) for amino acid analysis. A pooled quality control (QC) sample was prepared by mixing equal amounts (10 μl) of each extracted serum sample. Amino acids were separated using a C8 column (Rapid Resolution HT, 4.5 × 50 mm, 1.8 μm, Zorbax SB-C8) on a Agilent 1290 Infinity LC system (Agilent Technologies, CA, USA) coupled with quadrupole-ion trap mass spectrometer (QTRAP 5500, AB Sciex, DC, USA). Mobile phase A (10/90 Water/Acetonitrile) and Mobile phase B (90/10 Water/ Acetonitrile), both containing 10 mM of Ammonium formate, were used for chromatography separation. Acylcarnitine measurements were made using flow injection tandem mass spectrometry on the Agilent 6430 Triple Quadrupole LC/MS system (Agilent Technologies, CA, USA). The sample analysis was carried out at 0.4 ml/min of 80/20 Methanol/water as mobile phase, and injection of 4 µL of sample. Data acquisition and analysis were performed on Agilent MassHunter Workstation B.06.00 Software. Free and total L-carnitine analysis was carried out as previously described^16^. Data acquisition and analysis were performed on an Agilent MassHunter Workstation B.06.00 Software.

### Statistical methodology

Clinical characteristics are presented as mean and standard deviation (SD) for continuous data and frequency and percentage for categorical data. We analysed 83 metabolites comprising 65 acyl-carnitine metabolites, 16 amino acid metabolites and 2 carnitine metabolites. Metabolites with >25% of values below the lower limit of quantification were excluded from analysis (only C10:2 was excluded, hence a total of 83 metabolites were analyzed in the final sample). We normalized the distributions of all metabolites by a logarithmic transformation.

The association between clinical risk factors and LA function was assessed in 2 steps. First, simple linear regression with LA function as dependent variable was used individually. Further all clinical risk factors that show an association with *p* < 0.05 with LA function in univariate analysis were included in the multivariate linear regression respectively. In this analysis since central pulse pressure (PP) were highly correlated to central systolic blood pressure (SBP) (The Pearson correlation between central SBP and central PP is 0.81), we only included central SBP into the multivariable model if both central SBP and central PP are significant in the univariate analysis.

We identified amino acids associated with LA function, respectively, in 3 ways. Firstly, simple linear regression with LA function as a dependent variable was used respectively to determine the significance of the individual amino acids. Secondly, multivariate linear regression was conducted for each amino acids with *p* < 0.05 in univariate analysis adjusting for significant clinical risk factors identified. Thirdly, multivariate linear regression was conducted including all amino acids that show an association with *p* < 0.05 with LA function in the multivariate analysis adjusting for clinical confounders.

To identify metabolites correlations (65 acyl-carnitine metabolites and 2 carnitine metabolites) and reduce the dimensionality of correlated metabolites, we performed sparse principal component analysis (SPCA), which used a penalized matrix decomposition^17^. Comparing with the regular principal component analysis that suffers from the fact of a dense loading matrix from all variables, SPCA is capable of producing sparse loadings which makes it more biologically interpretable. Specifically, we set the orthogonality constraint^17^ on each component and the number of components to be 10. We reported the description on each component and the proportion of variance-accounted.

To assess the association between the 10 SPCA factors and LA function, we first performed simple linear regression with LA function as dependent variable, respectively. Further, for each SPCA factor, we performed multivariable linear regression adjusting for significant clinical confounders identified.

All statistical analyses were performed using STATA 13 (College Station, Texas, USA), while the SPCA and correlation matrix heatmap showing pairwise Pearson correlations (r) between amino acids and LA function were performed by R. For all analysis, a two-tailed *P* value of <0.05 was considered significant.

## Results

A total of 128 participants (mean age 70.5 ± 11.6 years; 52 women) were included in this analysis. All completed cardiac magnetic resonance imaging and had blood sample acquired on the same day. The majority of participants had vascular risk factors of hypertension (53.1%) and dyslipidemia (50.0%) while some had diabetes mellitus (21.9%). The central systolic and diastolic blood pressures of the participants were 139 ± 18 mmHg and 76 ± 11 mmHg respectively. Baseline clinical characteristics of the study sample are presented in Table 1. Additional CMR measurements of the left ventricle and echocardiogram-derived measurements are shown in Supplementary Table S2.

**Table 1:** Baseline clinical characteristics of study participants (N = 128).

Variable	Overall
Age (years)	70.5 (11.6)
Female	52 (40.6%)
Ever smoked	26 (20.3%)
Body mass index (kg/m^2^)	23.4 (3.1)
Hypertension	68 (53.1%)
Diabetes mellitus	28 (21.9%)
Dyslipidemia	64 (50.0%)
Heart rate (beats per minute)	74 (13)
Central systolic blood pressure (mmHg)	139 (18)
Central diastolic blood pressure (mmHg)	76 (11)
Central mean arterial pressure (mmHg)	102 (12)
Central pulse pressure (mmHg)	62 (17)
**Left atrial function**	
Reservoir strain (εs)	31.1 (7.8)
Conduit strain (εe)	13.2 (5.5)
Booster strain (εa)	16.6 (5.0)
Reservoir strain rate (SRs)	1.6 (0.5)
Conduit strain rate (SRe)	−1.4 (0.7)
Booster strain rate (SRa)	−2.2 (0.7)
Ratio of SRe/SRa	0.7 (0.5)

Mean (SD) are presented for continuous variables.

SRe/SRa = ratio of SRe over SRa.

We observed univariate associations between clinical variables and left atrial function. Age, central pulse pressure were significant for εs; age, ever smoking, hypertension, diabetes, dsylipidemia, central systolic blood pressure were significant for εe; heart rate was significant for εa; age, BMI, heart rate, central diastolic blood pressure, central pulse pressure were significant for SRs; age, ever smoking, hypertension, diabetes, dyslipidemia, central systolic blood pressure were significant for SRe; female gender, BMI, heart rate, central pulse pressure were significant for SRa; age, ever smoked, diabetes mellitus, dyslipidemia, central systolic blood pressure were significant for SRe/SRa.

Table 2 showed multivariate linear regression analysis between significant clinical variables and left atrial function, respectively. In multivariate analysis, age was significantly associated with εs (β = −0.30, p < 0.0001), εe (β = −0.3, p < 0.0001), SRs (β = −0.02, p < 0.0001), SRe (β = 0.04, p < 0.0001) and SRe/SRa (β = −0.01, p = 0.012). In contrast, only heart rate was significantly associated with εa (β = 0.1, p = 0.001) and SRa (β = −0.02, p < 0.0001). Except for diabetes mellitus that was associated with εe (β = −2.0, p = 0.032), hypertension, body mass index, dyslipidemia and gender were not associated with left atrial function.

**Table 2:** Multivariate analysis of clinical covariates associated with left atrial function.

Clinical covariates	LA function	Coef. (95% CI)	*P*-value
Age (years)	εs	−0.3 (−0.4, −0.1)	**<0.0001**
	εe	−0.3 (−0.4, −0.2)	**<0.0001**
	SRs	−0.02 (−0.03, −0.01)	**<0.0001**
	SRe	0.04 (0.04, 0.1)	**<0.0001**
	SRe/SRa	−0.01 (−0.02, −0.003)	**0.012**
Female	SRa	0.2 (−0.04, 0.5)	0.10
Ever smoked	εe	−1.2 (−3.0, −0.5)	0.16
	SRe	0.1 (−0.05, 0.3)	0.14
	SRe/SRa	−0.2 (−0.4, 0.04)	0.10
Body mass index ((kg/m^2^)	SRs	−0.02 (−0.05, 0.001)	0.063
	SRa	0.02 (−0.02, 0.1)	0.31
Hypertension	εe	0.1 (−1.5, 1.7)	0.91
	SRs	−0.01 (−0.2, 0.2)	0.89
	SRe	0.03 (−0.1, 0.2)	0.71
Diabetes mellitus	εe	−2.0 (−3.7, −0.2)	**0.032**
	SRe	0.2 (−0.003, 0.4)	0.054
	SRe/SRa	−0.2 (−0.4, 0.1)	0.14
Dyslipidemia	εe	0.2 (−1.3, 1.7)	0.79
	SRe	−0.04 (−0.2, 0.1)	0.62
	SRe/SRa	−0.1 (−0.3, 0.1)	0.29
Heart rate (beats per minute)	εa	0.1 (0.1, 0.2)	**0.001**
	SRs	0.006 (−0.001, 0.01)	0.080
	SRa	−0.02 (−0.03, −0.01)	**<0.0001**
Central systolic blood pressure (mmHg)	εe	−0.004 (−0.05, 0.04)	0.85
	SRe	0.001 (−0.003, 0.01)	0.61
	SRe/SRa	−0.004 (−0.01, 0.001)	0.12
Central diastolic blood pressure (mmHg)	SRs	0.01 (−0.0002, 0.01)	0.058
Central pulse pressure (mmHg)	εs	−0.03 (−0.1, 0.1)	0.56
	εe	−0.03 (−0.1, 0.05)	0.44
	SRs	0.001 (−0.004, 0.01)	0.69
	SRa	0.001 (−0.01, 0.01)	0.88

Variables were selected based on simple linear regression with *P* < 0.05 at univariate analysis. Univariable analysis results are described in the text.

Multiple regression models performed.

Outcome εs; Confounders Age, Central pulse pressure.

Outcome εe; Confounders Age, Ever smoked, Hypertension, Diabetes mellitus, Dyslipidemia, Central systolic blood pressure.

Outcome εa; Confounders Heart rate.

Outcome SRs; Confounders Age, Body mass index, Heart rate, Central diastolic blood pressure, Central pulse pressure.

Outcome SRe; Confounders Age, Ever smoked, Hypertension, Diabetes mellitus, Dyslipidemia, Central systolic blood pressure.

Outcome SRa; Confounders Female, Body mass index, Heart rate, Central pulse pressure.

Outcome SRe/SRa; Confounders Age, Ever smoked, Diabetes mellitus, Dyslipidemia, Central systolic blood pressure.

We analysed 83 metabolites comprising 65 acyl-carnitine metabolites, 16 amino acid metabolites and 2 carnitine metabolites. The list of measured metabolites is presented in supplementary Tables S3 to S4.

**Figure 2:**
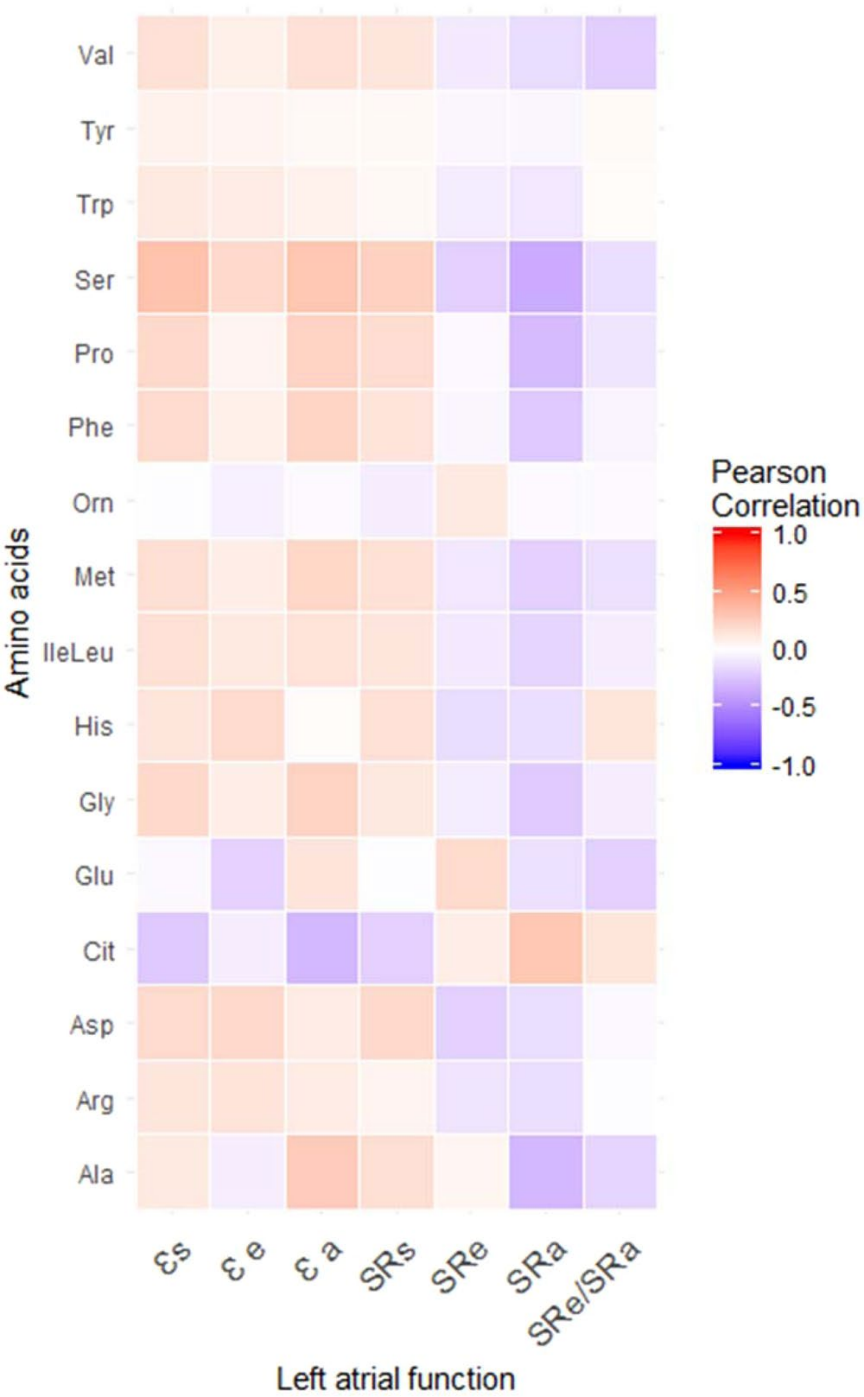
Heat map of correlations betweewas associated with ratio of conduitn amino acids and outcomes individually. The correlations increased from purple to red. Significant correlations are coloured while non-significant correlations are colourless).

Correlations for the 16 amino acids were assessed using the Pearson correlation analysis (Fig. 2). We observed serine was significantly correlated with all LA function except the ratio SRe/SRa (r ranges from −0.36 to 0.32; all *p* < 0.05) whilst arginine, histidine, ornithine, tryptophan and tyrosine were not correlated with any LA function. Table 3 shows multivariate analysis between individual amino acids and corresponding left atrial functions, adjusting for prior clinical covariates. Serine remains significantly associated with reservoir strain (*β* = 10.1; 95% CI 2.0, 18.2; *p* = 0.015), reservoir strain rate (*β* = 0.5; 95% CI 0.04, 1.0; *p* = 0.033) and booster strain rate (*β* = −0.9; 95% CI −1.7, −0.2; *p* = 0.016). Citrulline was associated with reservoir strain (*β* = −4.0; 95% CI −7.2, −0.7; p = 0.016), booster strain (*β* = −3.4; 95% CI −5.5, −1.2; *p* = 0.002) and booster strain rate (*β* = 0.4; 95% CI 0.1, 0.7; *p* = 0.019). Valine was associated with ratio of conduit strain rate to booster strain rate (*β* = −0.4; 95% CI −0.7, −0.02; *p* = 0.039).

**Table 3:** Multivariable model for association between individual amino acids and left atrial function.

Amino acids	LA function	Coef (95% CI)	*P*-value
Ala	εa	0.2 (−4.3, 4.7)	0.93
	SRa	−0.2 (−0.8, 0.5)	0.57
	SRe/SRa	−0.3 (−0.7, 0.04)	0.081
Arg	—	—	—
Asp	—	—	**—**
Cit	εs	−4.0 (−7.2, −0.7)	**0.016**
	εa	−3.4 (−5.5, −1.2)	**0.002**
	SRa	0.4 (0.1, 0.7)	**0.019**
Glu	—	—	**—**
Gly	εs	1.5 (−5.5, 8.6)	0.67
	εa	2.1 (−2.3, 6.6)	0.35
	SRa	−0.1 (−0.8, 0.6)	0.76
His	**—**	**—**	**—**
IleLeu	SRa	0.1 (−0.5, 0.8)	0.68
Met	εa	0.7 (−2.1, 3.5)	0.63
	SRa	−0.1 (−0.5, 0.4)	0.78
Orn	**—**	**—**	**—**
Phe	εs	5.1 (−1.8, 12.0)	0.14
	εa	3.8 (−0.9, 8.6)	0.11
	SRa	−0.6 (−1.3, 0.2)	0.13
Pro	εs	0.2 (−5.7, 6.1)	0.95
	εa	0.1 (−3.9, 4.2)	0.95
	SRs	0.2 (−0.1, 0.6)	0.20
	SRa	−0.2 (−0.8, 0.4)	0.56
Ser	εs	10.1 (2.0, 18.2)	**0.015**
	εa	4.5 (−0.8, 9.8)	0.098
	SRs	0.5 (0.04, 1.0)	**0.033**
	SRa	−0.9 (−1.7, −0.2)	**0.016**
Trp	—	—	—
Tyr	—	—	—
Val	SRe/SRa	−0.4 (−0.7, −0.02)	**0.039**

(1) The association of amino acids with LA function was first assessed using simple linear regression, individually. (2) For each amino acids with *p* < 0.05 in univariate analysis, multivariate linear regression was further conducted adjusting for clinical confounders. The adjustments are.

Outcome εs; Confounder age.

Outcome εe; Confounder Age, Diabetes mellitus.

Outcome εa; Confounder Heart rate.

Outcome SRs; Confounder Age.

Outcome SRe; Confounder Age.

Outcome SRa; Confounder Heart rate.

Outcome SRe/SRa; Confounder Age.

(3) Finally multivariate linear regression was conducted including all amino acids that show an association with *p* < 0.05 in the multivariable analysis (2).

Sparse principal component analysis identified 10 metabolite factors clustering in biologically related groupings (Table 4). Loading values for the 10 metabolite factors are illustrated in Supplementary Figure P. Univariate association between each of the 10 SPCA factors and left atrial function is shown in Supplementary Table S5. We noted only Factor 3 showed significant association with εs, εe, SRs, SRe and SRa in univariate analysis. However, after adjustments for significant clinical covariates Factor 3 (medium and long-chain dicarboxyl/hydroxyl acyl-carnitines) was only associated with reservoir strain (εs) (β = −0.6, *p* = 0.038) (Table 5).

**Table 4:** Factors identified by sparse principal component analysis and the associated individual components, description and variance.

Factors	Description	Components	Percentage of variance accounted
1	Medium and long-chain carnitines	C8, C8-DC, C12:1, C12, C12-OH/C10-DC, C14:2, C14:1, C14, C16:3, C16:2, C16:1, C18:1	11
2	Short chain dicarboxyl/hydroxyl carnitines	C3, C4, C5:1, C5, C4-OH, C6, C5OHC3DC, C4DCC6OH, C5DC, C81OHC61DC, C8OHC6DC, C103, C81DC, C8-DC	6.3
3	Medium and long chain dicarboxyl/hydroxyl carnitines	C81OHC61DC, C122OHC102DC, C121OH, C142OH, C141OH, C163OHC143DC, C162OH C183OHC163DC, C182OHC162DC, C201, C20, C202OHC182DC, C201OHC181DC, C20OHC18DC, C221	7.2
4	Long chain carnitines	C16, C183, C182, C181, C18, C204, C203, C202, C201, C202OHC182DC, C225, C224 C223	6.0
5	Medium and long chain dicarboxyl/hydroxyl carnitines	C4OH, C8OHC6DC, C8DC, C12OHC10DC, C141OH, C14OHC12DC, C162OH, C161OHC141DC, C16OH, C181OHC161DC, C18OHC16DC, C20, C201OHC181DC, C20OHC18DC	7.4
6	Wide spectrum carnitines including odd short chain carnitines	C2, C3, C51, C5, C5OHC3DC, C101, C7DC, C121, C12, C14, C142OH, C163, C162OH, C16OH, C183, C182, C18, C183OHC163DC, C182OHC162DC, C204, C203, C202, C201, C203OHC183DC, C225, C223, C222, C22, Free Carnitine, Total Carnitine	3.8
7	Wide spectrum carnitines including ketone-derived carnitine	C2, C4OH, C6, C81, C5DC, C81OHC61DC, C103, C101, C10, C81DC, C122, C143, C142, C14, C142OH, C14OHC12DC, C162, C161, C16, C162OH, C161OHC141DC, C183, C182, C183OHC163DC, C18OHC16DC, C204, C202, C201OHC181DC, C224, C222, C22	4.5
8	Wide spectrum carnitines including odd short chain carnitines	C3, C51, C4DCC6OH, C5DC, C81OHC61DC, C8OHC6DC, C7DC, C81DC, C8DC, C122, C121, C12OHC10DC, C14OHC12DC, C16, C16OH, C183OHC163DC, C18OHC16DC, C204, C201, C20OHC18DC, C224, C223, C222, C221, Free Carnitine, Total Carnitine	2.2
9	Wide spectrum carnitines including ketone-derived carnitine	C2, C51, C4OH, C6, C5OHC3DC, C81OHC61DC, C101, C81DC, C12, C122OHC102DC, C121OH, C14, C142OH, C14OHC12DC, C162, C183, C182, C181, C183OHC163DC, C182OHC162DC, C181OHC161DC, C18OHC16DC, C204, C203, C201OHC181DC, C20OHC18DC, C225, Free Carnitine, Total Carnitine	2.3
10	Medium and long chain carnitines	C10, C143, C142, C14, C143OHC123DC, C142OH, C163, C16, C181, C18, C182OHC162DC, C204, C203, C201, C20, C221, C22	2.3

**Table 5:** Linear regression on PCA factor 3 formed using acylcarnitine.

	Unadjusted Coef (95% CI)	*P*-value	Adjusted Coef (95% CI)	*P*-value
εs	−0.8 (−1.4, −0.2)	**0.006**	**−0.6 (−1.1, −0.03)**	**0.038**
εe	−0.5 (−0.9, −0.1)	**0.017**	−0.2 (−0.5, 0.1)	0.16
εa	−0.3 (−0.7, 0.04)	0.076	−0.3 (−0.6, 0.1)	0.18
SRs	−0.04 (−0.1, −0.01)	**0.026**	−0.03 (−0.1, 0.01)	0.14
SRe	0.1 (0.01, 0.1)	**0.014**	0.02 (−0.01, 0.1)	0.20
SRa	0.1 (0.002, 0.1)	**0.044**	0.04 (−0.01, 0.1)	0.13
SRe/SRa	0.01 (−0.03, 0.1)	0.63	0.03 (−0.01, 0.1)	0.19

εs, SRs, SRe, and SRe/SRa: age is adjusted; εa and SRa: heart rate is adjusted; εe: age and diabetes are adjusted;.

SRe/SRa = ratio of SRe over SRa.

Bold indicates significance at the 5% level.

## Discussion

Among community adults free of symptomatic cardiovascular disease, left atrial reservoir, conduit, and booster strain and strain rates were differentially associated with clinical variables and circulating metabolomics profiles. Age was independently associated with reservoir and conduit strain and strain rate, diabetes mellitus was associated with conduit strain, whereas heart rate was associated with booster strain and strain rate. Among the specific phases of left atrial function, serine was associated with reservoir function and booster strain rate, citrulline was associated with reservoir, booster and booster strain rate and valine was associated with ratio of conduit strain rate to booster strain rate. A combination of medium to long chain dicarboxyl acylcarnitines were associated with reservoir strain.

### Clinical Implications

This study advances the field of CMR feature tracking for future clinical applications. Previous studies have supported the concept that left atrial dilation and impairments in left atrial function may reflect the influence of concomitant risk factors^18^ rather than age alone. In our study consisting of adults with risk factors, age is seen in independent associations with left atrial function even in the presence of risk factors, emphasizing that age plays an important role in assessment of left atrial function.

Using real-world data obtained from a community cohort of aged adults, our results suggest that age was independently associated with reservoir and conduit strain and strain rate. Our observations concur with data demonstrated by Evin *et al*.^4^, who found similar decreases in left atrial reservoir and conduit phases with age, while also observing no association between booster strain and age. Our cohort included elderly subjects which were importantly lacking in the Evin *et al*.’s cohort. As such, our data nicely complement their findings, strengthening the observation that association between age and reservoir and conduit phases extends into older age groups. Taken together, future studies using reservoir and conduit strain and strain rates should therefore consider the critical role that age may play in influencing reservoir and conduit strain parameters. Age did not appear to influence booster functions.

We found that heart rate was independently associated with booster strain and booster strain rate. This is a novel finding not previously observed in similar asymptomatic cohorts, and deserves special attention, given the widespread use of medications that may influence heart rate in patients such as those with hypertension. Our observations about the association between heart rate and booster strain reflect similar concerns by a recent study that demonstrated impairments in booster strain associated with use of beta-blockers, among a hypertensive cohort^19^. While assessment for medication use such as beta-blockers might have sharpened our current observation regarding heart rate, which we did not have information for, our findings highlight the importance for future therapeutic trials to consider the impact of interventions that may have influence heart rate, particularly for improving booster function. Furthermore, booster strain and booster strain rate may be viewed as diagnostic markers of left atrial function that is independent of age (and risk factors), providing a unique opportunity for clinical applications to use them additionally as robust markers of disease progression less likely influenced by chronological aging. For instance, it has been shown that in the area of post-operative atrial fibrillation prediction, data from echocardiography have identified subclinical left atrial booster strain dysfunction in patients with severe aortic stenosis as a predictive marker of atrial fibrillation, regardless of global left ventricular function, left atrial volume index or aortic stenosis severity^20^. Subtle changes in booster strain function may be used as future marker of atrial fibrillation detection, which is particularly common among elderly populations^21^.

### Mechanistic Underpinnings

To the best of our knowledge, our study is the first to measure circulating metabolomics profiles in conjunction with left atrial function using the CMR feature tracking technique in a cohort without overt CVD (i.e., with preserved left ventricular function). This is in contrast to previous work that has studied metabolomics of the heart under conditions where the disease of interest involved impairments in left ventricular function^22,23^. The current work provides a unique opportunity to understand the association between atrial function and metabolomics profiles, which is otherwise difficult to demonstrate in disease states where the left ventricle is frequently abnormal. Our observations are further strengthened by adjustments for clinical variables, known to influence these phases of left atrial function.

Acyl-carnitines reflect upon mitochondrial fuel metabolism and changes in the pattern of individual acyl-carnitine species may reflect both global alterations in mitochondrial function as well as specific changes in patterns of fuel use. Alteration in long-chain acyl carnitines have previously been detected in symptomatic cohorts with clinical cardiovascular disease^9–12^, including high-risk elderly with coronary artery disease or stroke^13^. The findings underscore important associations between mitochondrial pathways and cardiovascular disease. The dicarboxyl and hydroxyl acyl-carnitines are a specific class of acyl-carnitines generated via omega- and alpha-oxidation. Changes in the dicarboxyl- and hydroxyl-carnitines thus may reflect alterations in pathways spanning the endoplasmic reticulum (ER)^24^, the peroxisome^25,26^ and mitochondria. Our study identifies a unique association between a combination of medium and long chain dicarboxyl carnitines and reservoir function. This finding highlights potential links between ER, peroxisomal and mitochondrial function and left atrial reservoir function.

In addition, we observed novel patterns between left atrial function and circulating amino acids. Serine is a glucogenic amino acid which can also contribute to the biosynthesis of nucleotides as well as the ceramides, important signalling intermediates which have been linked to the development of cardiovascular disease^27^. Our novel data demonstrates an association between circulating levels of serine with left atrial function preceding clinically manifest atrial disease. Our hypothesis-generating data (1) supports the emerging recognition of serine-related molecules in atrial-related function^28–31^; (2) demonstrates it for the first time in a pre-disease cohort; and (3) suggests that circulating levels of serine are significantly associated with larger magnitude (i.e., beneficial) of left atrial reservoir strain and strain rate. Longitudinal studies in the future may use circulating profiles of serine to further investigate associations from a phase of pre-disease, atrial remodelling to clinical atrial dysfunction and disease.

We found that citrulline was associated with reservoir strain and booster strain rate. Citrulline contributes to the urea cycle, a mitochondrial-based pathway which has been reported to be involved in CVD^32^. Citrulline is also a major component of the nitric oxide pathway, which has been heavily implicated in the development of CVD^33^ and may be important in atrial dysfunction^34^. Finally, valine was associated with ratio of conduit strain rate to booster strain rate. Valine is a branched-chain amino acid and has been consistently found to be associated with the development of insulin resistance and type 2 diabetes^35,36^. Changes in the branched-chain amino acids have also been linked to cardiovascular disease^11^. While the link between branched chain amino acids and disease is still unclear, evidence suggests that it contributes to altered mitochondrial function^35^. These results further highlight the potential importance of mitochondrial fuel metabolism changes in the pathogenesis of altered atrial function.

### Study limitations

While our study was prospective, sample size was relatively small although we were able to identify statistically significant associations within the group. We acknowledge that samples obtained in a non-fasting state may potentially introduce analytic differences in post-absorptive states between the subjects studied. As a community-based driven study, we recognize challenges in getting subjects to fast while participating in these studies. Future studies comprising of fasting samples may provide additional insights as to the effect of fasting on similar analyses.

While we corrected for available clinical factors, we cannot exclude the possibility that additional factors that were not included could have influenced our findings. Our study design is cross-sectional and hence we cannot infer causal relationships. Future longitudinal follow-up in a larger cohort may provide greater power as well as further insights as to causality. However, our results highlight the clinical relevance of pursuing future clinical investigations using both a clinical imaging and a molecular approach as such a novel approach may help identify mechanisms involved in cardiovascular diseases in specific cohorts^37^.

## Conclusion

The different phases of left atrial function as measured by CMR feature tracking were differentially associated with clinical and circulating metabolite factors. Our results emphasize the need to appreciate the impact of age and heart rate separately on phases of reservoir, conduit and booster functions. Furthermore, our results highlight potentially important upstream metabolic perturbations involving mitochondrial fuel metabolism which may result in changes to the ceramide pathway, urea cycle as well as nitric oxide metabolism.

## Electronic supplementary material

Supplementary Data
