# Supplementary material for: Dissecting Clinical and Metabolomics Associations of Left Atrial Phasic Function by Cardiac Magnetic Resonance Feature Tracking
Source: Sci Rep. 2018 May 25;8:8138. doi: 10.1038/s41598-018-26456-8 (PMC5970174; doi:10.1038/s41598-018-26456-8)
Supplement: Supplementary file 1 — Supplementary Data [file 41598_2018_26456_MOESM1_ESM.doc]

**Dissecting Clinical and Metabolomics Associations of Left Atrial Phasic Function by Cardiac Magnetic Resonance Feature Tracking**

Angela S.Koh^1,2^,MBBS,MPH; Fei Gao^1,2^,PhD; Shuang Leng^1^,PhD; Jean-Paul Kovalik^2,3^,MD, PhD; Xiaodan Zhao^1^, PhD; Ru San Tan^1,2^,MBBS; Kevin Timothy Fridianto^2^; Jianhong Ching^2^, PhD; Serene JM Chua^1^; Jian-Min Yuan^5,6^, MD, PhD; Woon-Puay Koh^2,4^,MBBS, PhD; Liang Zhong^1,2^,PhD

Affiliations:

1. National Heart Centre Singapore, Singapore
2. Duke-NUS Medical School, Singapore
3. Department of Endocrinology, Singapore General Hospital
4. Saw Swee Hock School of Public Health, National University of Singapore, Singapore
5. Division of Cancer Control and Population Sciences, University of Pittsburgh Cancer Institute, Pittsburgh, PA, USA
6. Department of Epidemiology, Graduate School of Public Health, University of Pittsburgh, Pittsburgh, PA, USA

Corresponding author:

Angela S. Koh

National Heart Centre Singapore

5 Hospital Drive, Singapore 169609

Email: [angela.koh.s.m@nhcs.com.sg](mailto:angela.koh.s.m@nhcs.com.sg)

Telephone: +65 6704 8961

Fax: +65 6222 9258

**Supplementary Data**

**Supplementary Figure S1a: Bland Altman plots for inter-observer variability obtained for strain and strain rate**


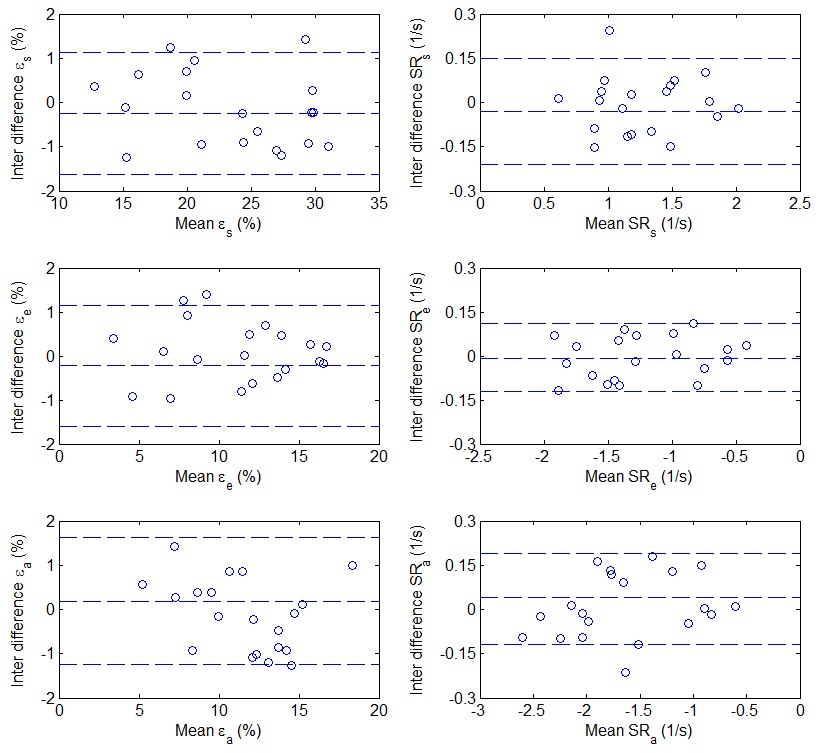


**Supplementary Figure S1b: Bland Altman plots for intra-observer variability obtained for strain and strain rate**

**
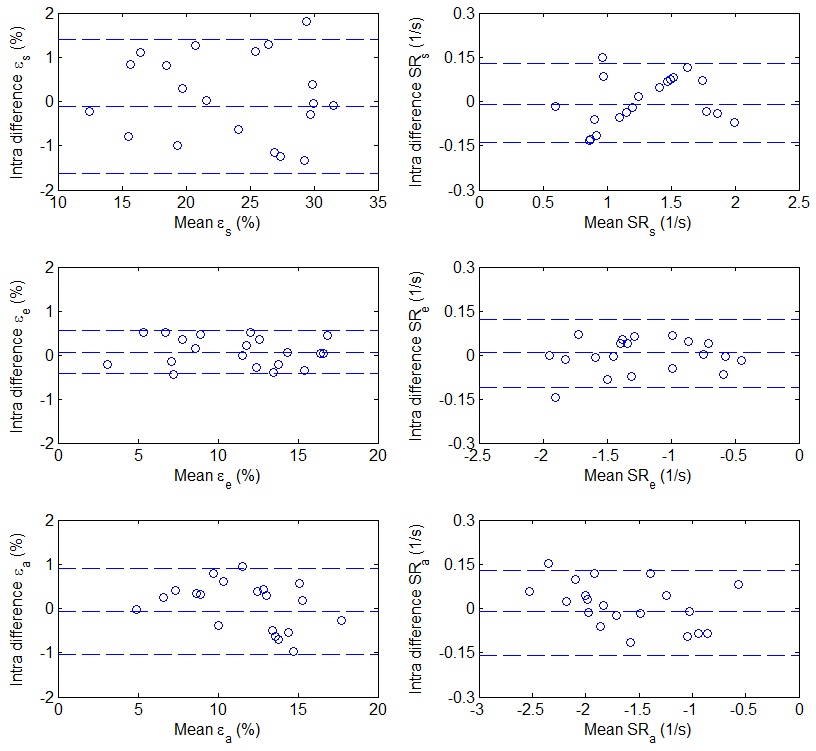
**

Volumetric analysis was performed using commercial software (QMass, Medis, Leiden, The Netherland). LA volume in this study was calculated as 0.85 × 4-chamber area × 2-chamber area/average of the 2 lengths [1]. LA volumes were evaluated at left ventricular end-systole (LAV max), at left ventricular diastole prior to LA contraction (LAV pre-a) and at left ventricular end-diastole (LAV min). The following equations were used to quantify total LA emptying fraction (LAEF Total, corresponding to reservoir function), passive LA emptying fraction (LAEF Passive, corresponding to conduit function) and active LA emptying fraction (LAEF Active, corresponding to booster pump function) [2]:


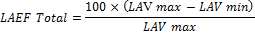


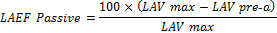


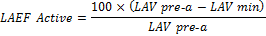


1. Taggart NW, Cetta F, O'Leary PW, Seward JB, Eidem BW. Left atrial volume in children without heart disease and in those with ventricular septal defect or patent ductus arteriosus or hypertrophic cardiomyopathy. Am J Cardiol. 2010;106(10):1500-1504.
2. Kowallick JT, Kutty S, Edelmann F, Chiribiri A, Villa A, Steinmetz M et al. Quantification of left atrial strain and strain rate using Cardiovascular Magnetic Resonance myocardial feature tracking: a feasibility study. J Cardiovasc Magn Reson. 2014;16:60.

Good correlation was observed between volumetric measurements and the CMR-derived strain and strain rate parameters for all LA phasic functions (Supplementary Table S1).

Supplementary table S1. Correlation of left atrial volumetric measurements and corresponding CMR-derived strain and strain rate parameters

| **Left atrial function** | **Strain & strain rates** | **Volumetric measurements** | **Correlation coefficient** | **P value** |
| --- | --- | --- | --- | --- |
| **Reservoir** | ε_s_ | LAEF Total | 0.84 | < 0.001 |
|  | SR_s_ | LAEF Total | 0.71 | < 0.001 |
| **Conduit** | ε_e_ | LAEF Passive | 0.84 | < 0.001 |
|  | SR_e_ | LAEF Passive | -0.78 | < 0.001 |
| **Booster pump** | ε_a_ | LAEF Active | 0.78 | < 0.001 |
|  | SR_a_ | LAEF Active | -0.67 | < 0.001 |

ε: strain; SR: strain rate; LAEF: left atrial emptying fraction

CMR feature tracking analysis was conducted on 30 randomly selected cases employing recently introduced QStrain software (version 2.0, Medis, Leiden, The Netherlands). The long-axis 4- and 2-chamber views were used to derive left atrial global longitudinal strain values. For the feature tracking analysis, endocardial borders were manually delineated in end-diastolic frame followed by automatic tracking of the endocardial contours by the software throughout the cardiac cycle.

Our fast assessable strain indexes showed good correlation with the strain values from the QStrain software (r = 0.85, 0.85 and 0.73 for ɛ_s_, ɛ_e_, and ɛ_a_) with small bias. The corresponding regression lines and Bland-Altman plots are shown in Supplementary Figure A.

**Supplementary Figure A**


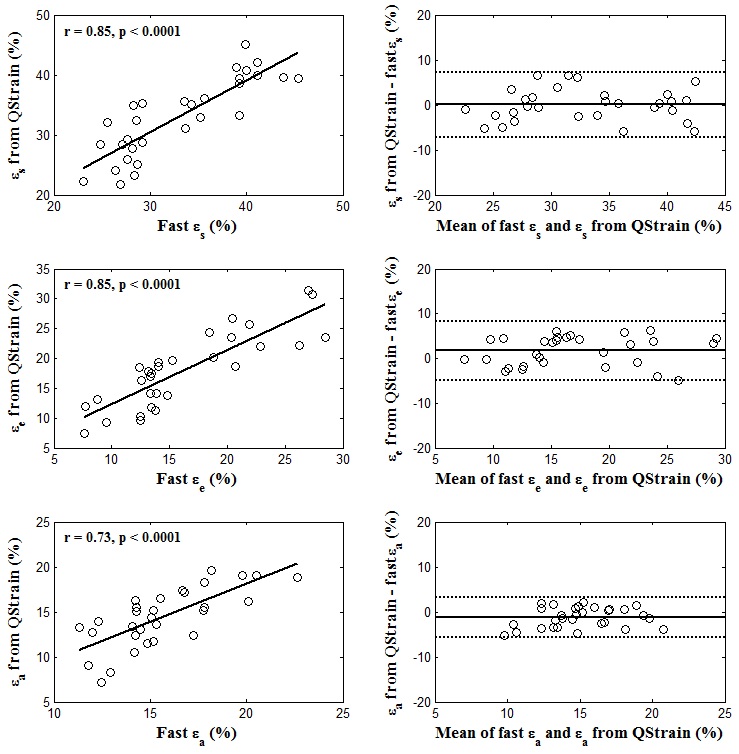


Supplementary Figure A. Regression and Bland-Altman plots between fast assessable strain indexes and the strain values obtained from commercial software.

**Supplementary table S2: Other cardiac measurements of the cohort**

| **CMR measurements** | **Mean (SD)** |
| --- | --- |
| LV mass (g) | 75.0 (20.0) |
| LV mass index (g/m^2^) | 46.6 (11.1) |
| LVEDV (ml) | 106.7 (27.9) |
| LVESV (ml) | 37.1 (17.3) |
| LV stroke volume (ml) | 69.3 (15.7) |
| LV ejection fraction (%) | 65.5 (7.3) |
| **Echocardiogram-derived measurements** |  |
| Left atrial volume index (ml/m^2^) | 23.6 (8.1) |
| MV E peak (ms) | 0.7 (0.2) |
| MV A peak (ms) | 0.8 (0.2) |
| E/A (ratio) | 0.9 (0.3) |
| Mitral deceleration time (ms) | 210.0 (37.0) |
| PASP (mmHg) | 27.6 (6.5) |
| PVS (cm/s) | 57.7 (11.5) |
| PVD (cm/s) | 47.5 (14.7) |
| PVA (cm/s) | 28.9 (5.2) |
| Septal Sm (m/s) | 0.1 (0.01) |
| Septal Em (m/s) | 0.1 (0.02) |
| Septal Am (m/s) | 0.1 (0.02) |
| Lateral Sm (m/s) | 0.1 (0.02) |
| Lateral Em (m/s) | 0.1 (0.02) |
| Lateral Am (m/s) | 0.1 (0.02) |

Left ventricle (LV); end-diastolic volume (EDV); mitral valve (MV); peak blood velocity from Doppler echocardiography at early filling phase (E) and at atrial contraction phase (A); PASP (pulmonary artery systolic pressure); pulmonary vein blood velocity at systolic phase (PVS), diastolic phase (PVD) and atrial reversal phase (PVA); myocardial velocity from tissue Doppler imaging at systolic phase (Sm), early filling phase (Em) and atrial contraction phase (Am)

**Supplementary table S3: List of measured metabolites**

| **Short name** | **Name** |
| --- | --- |
| Ala | Alanine |
| Arg | Arginine |
| Asp | Aspartic acid |
| Cit | Citrulline |
| Glu/Gln | Glutamate/Glutamine |
| Gly | Glycine |
| His | Histidine |
| Ile/Leu | Leucine/Isoleucine |
| Met | Methionine |
| Orn | Ornithine |
| Phe | Phenylalanine |
| Pro | Proline |
| Ser | Serine |
| Trp | Tryptophan |
| Tyr | Tyrosine |
| Val | Valine |
| C2 | Acetyl carnitine |
| C3 | Propionyl carnitine |
| C4 | Butyryl carnitine or isobutryl carnitine |
| C5:1 | Tiglyl carnitine or 3-methyl crotonyl carnitine |
| C5 | Isovaleryl, 3-methylbutyryl carnitine , 2-Methylbutyryl, valeryl or pivaloyl carnitine |
| C4-OH | D-3-Hydroxy-butyryl carnitine, L-3-hydroxybutyryl carnitine |
| C6 | Hexanoyl carnitine |
| C5-OH/C3-DC | 3-Hydroxy-isovaleryl carnitine or malonyl carnitine |
| C4-DC/C6-OH | Methylmalonyl carnitine or succinyl carnitine |
| C8:1 | Octenoyl carnitine |
| C8 | Octanoyl carnitine |
| C5-DC | Glutaryl carnitine, ethylmalonyl carnitine |
| C8:1-OH/C6:1-DC | 3-Hydroxy- octenoyl carnitine or hexenedioyl carnitine |
| C8-OH/C6-DC | 3-hydroxy octanoyl carnitine or adipoyl carnitine, 3-methylglutaryl carnitine |
| C10:3 | Decatrienoyl carnitine |
| C10:1 | Decenoyl carnitine |
| C10 | Decanoyl carnitine |
| C7-DC | Pimeloyl carnitine, heptanedioyl carnitine |
| C8:1-DC | Octadecenedioyl carnitine |
| C8-DC | Suberoyl carnitine |
| C12:2 | - |
| C12:1 | Dodecenoyl carnitine |
| C12 | Lauroyl carnitine |
| C12:2-OH/C10:2-DC | - |
| C12:1-OH | Hydroxydodecenoyl carnitine |
| C12-OH/C10-DC | 3-Hydroxy-dodecanoyl carnitine or sebacoyl carnitine |
| C14:3 | - |
| C14:2 | Tetradecadienoyl carnitine |
| C14:1 | Tetradecenoyl carnitine |
| C14 | Myristoyl carnitine |
| C14:3-OH/C12:3-DC | - |
| C14:2-OH | 3-Hydroxytetradecenoylcarnitine |
| C14:1-OH | 3-Hydroxy-tetradecenoyl carnitine |
| C14-OH/C12-DC | 3-Hydroxy-tetradecanoyl carnitine or dodecanedioyl carnitine |
| C16:3 | - |
| C16:2 | Hexadecadienoyl carnitine |
| C16:1 | Palmitoleoyl carnitine |
| C16 | Palmitoyl carnitine |
| C16:3-OH/C14:3-DC | - |
| C16:2-OH | 3-Hydroxyhexadecadienoyl carnitine |
| C16:1-OH/C14:1-DC | 3-Hydroxy-palmitoleoyl carnitine or cis-5-tetradecenedioyl carnitine |
| C16-OH | 3-Hydroxy-hexadecanoyl carnitine |
| C18:3 | Linolenyl carnitine |
| C18:2 | Linoleyl carnitine |
| C18:1 | Oleyl carnitine |
| C18 | Stearoyl carnitine |
| C18:3-OH/C16:3-DC | 3-Hydroxyl-linolenyl carnitine or |
| C18:2-OH/C16:2-DC | 3-Hydroxy-linoleyl carnitine or hexadecadienedioyl carnitine |
| C18:1-OH/C16:1-DC | 3-Hydroxy-octadecenoyl carnitine or hexadecanedioyl carnitine |
| C18-OH/C16-DC | 3-Hydroxy-octadecanoyl carnitine or hexadecanedioyl carnitine, thapsoyl carnitine |
| C20:4 | Arachidonoyl carnitine |
| C20:3 | Dihomogammalinolenyl carnitine |
| C20:2 | - |
| C20:1 | - |
| C20 | Arachidoyl carnitine, eicosanoyl carnitine |
| C20:3-OH/C18:3-DC | - |
| C20:2-OH/C18:2-DC | - |
| C20:1-OH/C18:1-DC | Octadecenedioyl carnitine |
| C20-OH/C18-DC | 3-Hydroxy-eicosanoyl carnitine or octadecanedioyl carnitine |
| C22:5 | - |
| C22:4 | - |
| C22:3 | - |
| C22:2 | - |
| C22:1 | - |
| C22 | Docosanoyl carnitine, Behenoyl carnitine |
| Free Carnitine |  |
| Total Carnitine |  |

**Supplementary table S4:** Summary of amino acids

| **Metabolites** | **Mean (SD) μm** |
| --- | --- |
| Ala | 495.1 (136.6) |
| Arg | 116.4 (27.6) |
| Asp | 23.2 (6.2) |
| Cit | 34.4 (13.9) |
| Glu | 93.4 (23.5) |
| Gly | 233.5 (49.5) |
| His | 77.8 (22.6) |
| IleLeu | 151.5 (45.6) |
| Met | 27.2 (10.1) |
| Orn | 86.2 (28.3) |
| Phe | 77.3 (15.7) |
| Pro | 255.6 (72.2) |
| Ser | 122.4 (24.1) |
| Trp | 55.1 (14.1) |
| Tyr | 71.8 (21.2) |
| Val | 246.1 (61.5) |

**Supplementary Figure P**

1. **Sparse PCA loading values for Factor 1 (PC1)**

1. **Sparse PCA loading values for Factor 2 (PC2)**

1. **Sparse PCA loading values for Factor 3 (PC3)**

1. **Sparse PCA loading values for Factor 4 (PC4)**

1. **Sparse PCA loading values for Factor 5 (PC5)**

1. **Sparse PCA loading values for Factor 6 (PC6)**

1. **Sparse PCA loading values for Factor 7 (PC7)**

1. **Sparse PCA loading values for Factor 8 (PC8)**

1. **Sparse PCA loading values for Factor 9 (PC9)**

1. **Sparse PCA loading values for Factor 10 (PC10)**

**Supplementary table S5: Coefficient and 95% confidence generated using linear regression on 10 PCA formed using acylcarnitines with left atrial function**

|  | **Ɛs** | **Ɛe** | **Ɛa** | **SRs** | **SRe** | **SRa** | **SRe/SRa** |
| --- | --- | --- | --- | --- | --- | --- | --- |
| Factor 1 | 0.3 (-0.2, 0.8) | 0.3 (-0.1, 0.6) | 0.1 (-0.3, 0.4) | 0.02 (-0.01, 0.05) | -0.03 (-0.1, 0.02) | -0.01 (-0.1, 0.04) | 0.01 (-0.03, 0.04) |
| Factor 2 | -0.1 (-0.8, 0.6) | -0.2 (-0.6, 0.3) | -0.03 (-0.5, 0.4) | 0.003 (-0.04, 0.05) | 0.03 (-0.03, 0.1) | 0.02 (-0.05, 0.1) | 0.01 (-0.04, 0.1) |
| Factor 3 | **-0.8 (-1.4, -0.2)** | **-0.5 (-0.9, -0.1)** | -0.3 (-0.7, 0.04) | **-0.04 (-0.1, -0.01)** | **0.1 (0.01, 0.1)** | **0.1 (0.002, 0.1)** | 0.01 (-0.03, 0.1) |
| Factor 4 | -0.1 (-0.7, 0.6) | -0.1 (-0.5, 0.4) | -0.1 (-0.5, 0.3) | 0.004 (-0.04, 0.04) | 0.01 (-0.05, 0.1) | 0.01 (-0.05, 0.1) | 0.002 (-0.04, 0.05) |
| Factor 5 | -0.2 (-0.8, 0.4) | -0.3 (-0.7, 0.1) | 0.1 (-0.2, 0.5) | 0.003 (-0.03, 0.04) | 0.04 (-0.01, 0.1) | -0.02 (-0.1, 0.03) | -0.03 (-0.1, 0.01) |
| Factor 6 | -0.3 (-1.1, 0.6) | -0.2 (-0.8, 0.4) | -0.02 (-0.6, 0.5) | -0.03 (-0.08, 0.02) | 0.03 (-0.05, 0.1) | -0.002 (-0.1, 0.1) | -0.002 (-0.1, 0.1) |
| Factor 7 | 0.3 (-0.5, 1.0) | -0.1 (-0.6, 0.4) | 0.3 (-0.2, 0.8) | 0.02 (-0.03, 0.07) | 0.02 (-0.05, 0.1) | -0.05 (-0.1, 0.02) | -0.02 (-0.1, 0.03) |
| Factor 8 | 0.5 (-0.7, 1.7) | 0.1 (-0.7, 0.9) | 0.2 (-0.6, 0.9) | 0.02 (-0.05, 0.1) | -0.03 (-0.1, 0.1) | -0.01 (-0.1, 0.1) | 0.002 (-0.1, 0.1) |
| Factor 9 | -0.03 (-1.1, 1.2) | 0.7 (-0.1, 1.5) | -0.5 (-1.3, 0.2) | 0.02 (-0.1, 0.1) | -0.1 (-0.2, 0.03) | 0.1 (-0.03, 0.2) | 0.06 (-0.02, 0.1) |
| Factor 10 | 0.8 (-0.2, 1.8) | 0.5 (-0.2, 1.2) | 0.4 (-0.2, 1.0) | 0.02 (-0.05, 0.1) | -0.1 (-0.1, 0.02) | -0.1 (-0.1, 0.04) | 0.04 (-0.03, 0.1) |

Bold indicates significance at the 5% level.
